# Corneal opacification, an atypical presentation of cystic fibrosis: a case report and review of the literature

**DOI:** 10.1186/s13256-022-03410-x

**Published:** 2022-05-12

**Authors:** Nazanin Farahbakhsh, Neda Bagherian, Sajad Shabanpourhaghighi, Soheila Khalilzadeh, Seyed Ahmad Tabatabaii, Ghamartaj Khanbabaee

**Affiliations:** 1 Department of Pediatric Pulmonology, Mofid Pediatrics Hospital, Shahid Beheshti University of Medical Sciences, Tehran, Iran; 2 School of Medicine, Shiraz University of Medical Sciences, Shiraz, Iran; 3 School of Medicine, Jahroom University of Medical Sciences, Jahroom, Iran; 4 Department of Pediatrics, Shahid Beheshti University of Medical Sciences, Tehran, Iran

**Keywords:** Cystic fibrosis, Corneal xerosis, Corneal opacity, Atypical

## Abstract

**Background:**

Respiratory and gastrointestinal manifestations are the main causes of mortality and morbidity in cystic fibrosis. Although these symptoms are well recognized, ophthalmic involvement of cystic fibrosis secondary to vitamin A deficiency is uncommon and has been reported very rarely in the medical literature.

**Case presentation:**

Here, we report a 2.5-year-old Iranian boy who presented with bilateral corneal xerosis and corneal opacity secondary to vitamin A deficiency related to cystic fibrosis malabsorption.

**Conclusion:**

Malabsorption of fat-soluble vitamins is a common presentation in cystic fibrosis, but corneal opacity secondary to vitamin A deficiency as the initial presentation of cystic fibrosis is a very rare manifestation of fat malabsorption. This highlights the importance of complete systemic examination besides ophthalmic examination in approaching a child with ophthalmic complaint.

## Introduction

Cystic fibrosis (CF) is the most common autosomal recessive disease among white people, having a broad spectrum of presentations as well as life-threatening complications. The most common presentations include respiratory and gastrointestinal symptoms [1]. Furthermore, CF affects the pancreas, especially the exocrine part, so patients are at risk of deficiency of both fat and fat-soluble vitamins [1]. Vitamin A deficiency is a common manifestation in advanced CF patients, but ocular manifestation of vitamin A deficiency as the initial manifestation of CF in young children is very rare [2, 3]. In this report, we describe a 2.5-year-old male patient presenting with corneal opacity who was diagnosed with CF. To the best of the authors’ knowledge, this is the first case of CF with respiratory symptoms and vitamin A deficiency presenting with corneal opacity.

## Case presentation

**Fig. 1:**
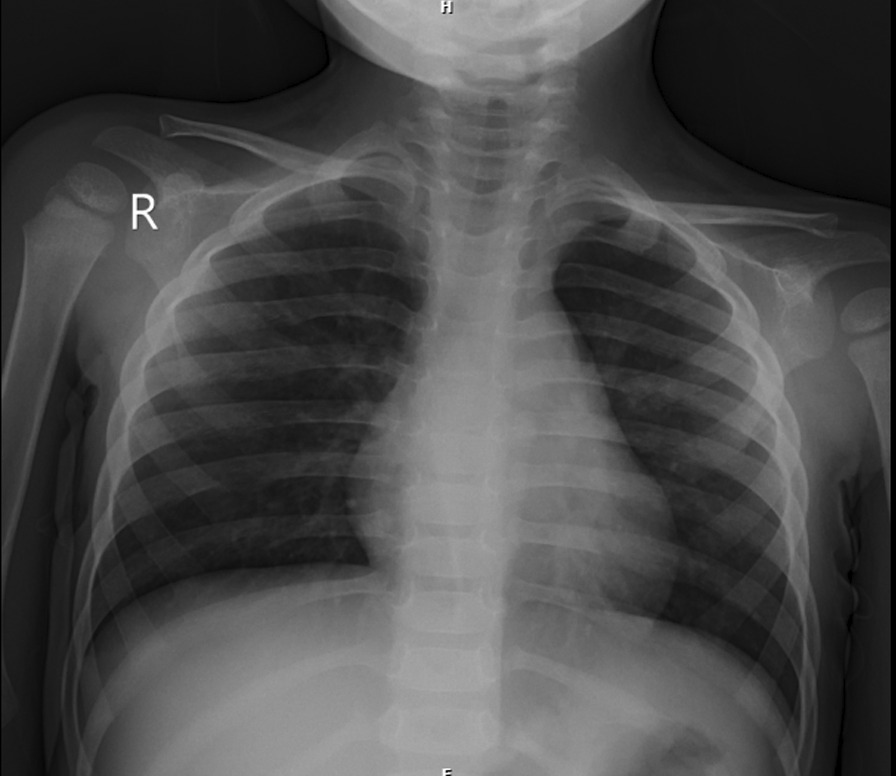
Chest X-ray

A 2.5-year-old Iranian boy from consanguineous marriage was referred to our center on December 2020 due to labored breathing, wet cough, and poor appetite 4 days prior to admission. Physical examination showed fever (T: 39 °C), tachypnea (RR: 40/minute), and tachycardia (120/minute) with prolonged expiration time, subcostal chest wall retraction, and bilateral diffused coarse crackle. Patient weight was 10.5 kg, and height was 91 cm (below 50th weight-for-height percentile). Although the patient had good appetite and was from a family with good socioeconomic status, he still showed failure to thrive. Neurological examination on admission was normal. Chest X-ray revealed hyperaeration, prebronchial cuffing, and bilateral nonspecific infiltrations in the lung (Fig. 1). He had a significant past medical history of admission several times due to pneumonia since early infancy and one pediatric intensive care unit (PICU) admission due to severe pneumonia. He had bilateral corneal opacity, which was diagnosed several months prior to this admission in another medical center. The etiology for corneal opacity was not investigated, but he was a candidate for corneal grafting. Ophthalmic examination in our center revealed bilateral severe corneal xerosis, total stromal haziness, normal intraocular pressure, and normal fundoscopy. Treatment with intravenous fluid, IV antibiotic (ceftriaxone), and antipyretic (paracetamol) was started. Since the patient had a prolonged expiration time with hyperaeration on chest X-ray, albuterol (MDI) was started with two puffs every 4 hours via spacer, to which he responded well. Initial laboratory tests on admission were as following: complete blood count (CBC); WBC 16,400/µl, 27% lymphocytes and 52% neutrophils, hemoglobin: 10 mg/dL, platelet count: 324,000/μL. C-reactive protein (CRP) 50 mg/l, urea 16 mg/dl (16.6–40), creatinine 0.6 mg/dl (0.2–0.8), and urine analysis was normal, Microscopic evaluation of the patient’s stool showed moderate fatty acid droplets. Culture samples taken from blood and throat were negative. Further investigations for the etiology of recurrent pneumonia were started including immunoglobulin (IG) level, CD flow cytometry, purified protein derivative (PPD), gastric washing for tuberculosis (TB), nontuberculous mycobacteria (NTM), and sweat chloride test, all being in the normal range except for sweat chloride level, which was 80 and 95 on two different days. Diagnosis of cystic fibrosis was confirmed, and appropriate treatment including pancreatic enzymes and multivitamins was started. Serum levels for vitamin A and D were 25.2 mg/dl (30−60 mg/dl) and 9 ng/dl (30–70) respectively, both at deficiency level. We raised the possibility that the patient’s corneal opacity could be due to profound vitamin A deficiency, so appropriate treatment with a single oral dose of 200,000 IU vitamin A followed by 1500 IU per day was started.

### Follow-up

The patient was discharged with standard CF treatment include multivitamin, azithromycin, pancreatic enzymes (CREON capsule), and routine outpatient visits to the CF clinic every month, plus ophthalmologist visits every 2 months. The serum level of vitamin A and D was checked 6 months after discharge, both being normal. Corneal opacity decreased gradually in outpatient follow-ups.

## Discussion

We report a 2.5-year-old boy as an undiagnosed case of CF with pulmonary involvement and ophthalmic manifestation secondary to vitamin A malabsorption, which is a rare complication of CF. The clinical manifestations of CF are vast and varied, depending on the organ being affected. Respiratory symptoms are the main cause of mortality and morbidity in these patients. Patients may present with typical CF-related symptoms such as malabsorption or chronic respiratory infections, which may enable earlier diagnosis. On the other hand, those with atypical disease, such as congenital absence, nasal polyps, pancreatitis, absence of the vas deferens, and azoospermia, may have delayed diagnosis [1]. The index case had respiratory symptoms, typical CF-related symptoms, but the corneal opacity was his parents’ major concern which had led them to ignore other symptoms in their child and delayed diagnosis.

About 85% of CF patients suffer from pancreatic insufficiency, which predisposes them to reduced enterohepatic circulation of bile acids leading to malabsorption of both fat and fat-soluble vitamins (A, D, E, K) [2]. Therefore, due to vitamin A deficiency, eye symptoms in CF are common and often subclinical, including xerophthalmia, nyctalopia, papilledema, and retinal hemorrhage [3]. Night blindness (nyctalopia) is the earliest and most common symptom of vitamin A deficiency [3]. Joshi *et al*. reported a newly diagnosed case of CF in a teenage boy with a chief complaint of night blindness due to vitamin A deficiency without any respiratory symptoms. As the patient had no respiratory symptoms, the diagnosis of CF was delayed until adolescence [4]. We report herein on a 2.5-year-old child with corneal opacity and failure to thrive, who had recurrent respiratory symptoms. As the patient had good response to bronchodilator, the patient’s respiratory symptoms were mistaken as bronchial hyperreactivity and no further investigations were carried out.

The first report of a case with xerophthalmia and corneal stromal edema with opacification was in 1989 by Lindenmuth *et al*., in a 16-month-old infant with severe photophobia and FTT but no respiratory symptoms, diagnosed later with CF. This highlights the unusual presentation of fat malabsorption as one of the main presentations of CF patients [5]. Wamsley *et al.* reported on a 5-month-old infant girl with FTT, pneumonia, and advanced keratomalacia, later diagnosed with CF [6]. Compared with the index case, the symptoms were more severe and started earlier, which may be due to the variety of genotypes and phenotypes of CF. In 2012, an 11-month-old infant was reported with corneal opacity and FTT but no respiratory symptoms, being diagnosed as CF with vitamin A deficiency [7]. The index case had typical pulmonary CF symptoms along with corneal opacity, which resolved after pancreatic enzyme replacement and multivitamin supplementation.

## Conclusion

The majority of patients with CF suffer from pancreatic insufficiency, which predisposes them to reduced enterohepatic circulation of bile acids leading to malabsorption of fat and fat-soluble vitamins (A, D, E, and K). This is the second report of a patient with CF and corneal opacity, which indicates the importance of complete systemic examination besides ophthalmic examination in approaching a child with ophthalmic complaint.

## Data Availability

The datasets used during the current study are available from the corresponding author on reasonable request.

## Abbreviations

CF: Cystic fibrosis
IG: Immunoglobulin
PPD: Purified protein derivative
TB: Tuberculosis
NTM: Nontuberculous mycobacteria
FTT: Failure to thrive
